# Missed diagnosis of giant hydronephrosis mimicking abdominal lymphatic malformation or omental cyst in a 16-year-old girl: A case report

**DOI:** 10.1016/j.ijscr.2025.111533

**Published:** 2025-06-19

**Authors:** Hyung Jun Kwon, Jinyoung Park

**Affiliations:** Department of Surgery, School of Medicine, Kyungpook National University, Kyungpook National University Hospital, Daegu, South Korea

**Keywords:** Hydronephrosis, Abdominal cyst, case report

## Abstract

**Introduction and importance:**

Giant hydronephrosis (GH) is an uncommon urological disease most often identified and managed during infancy or childhood. Diagnosing GH prior to surgical intervention can be difficult, resulting in potential misdiagnoses as ovarian tumors or intraabdominal cystic masses. Management of GH depends on the underlying pathology and the renal function of the affected kidney.

**Case presentation:**

A 16-year-old girl presented to local clinics with a 1-month history of abdominal distention and irregular menstruation. Abdominal computed tomography revealed a 33-cm cystic mass occupying the entire abdomen and displacing the bowel posteriorly, suggesting a lymphatic malformation or an omental cyst. The patient underwent emergent exploratory laparotomy. A 33-cm cystic mass originating from the right retroperitoneum was discovered and removed without complications. Histopathologic examination showed atrophied renal parenchyma and a dilated renal pelvis, confirming GH. The patient's postoperative course was uneventful, and she was discharged on postoperative day 7 in good condition.

**Clinical discussion:**

GH is a rare urological condition that is difficult to diagnose preoperatively, often leading to potential misdiagnoses.

**Conclusion:**

Although GH is exceedingly rare in the pediatric population, we recommend including it in the differential diagnosis of large intraabdominal cystic masses in pediatric patients.

## Highlights

• Diagnosing giant hydronephrosis (GH) prior to surgical intervention can be difficult, resulting in potential misdiagnoses as ovarian tumors or intraabdominal cystic masses.
• Management of GH depends on the underlying pathology and the renal function of the affected kidney.
• Although GH is exceedingly rare, it should be included in the differential diagnosis of large intraabdominal cystic masses in pediatric patients.

## 1 Introduction

Giant hydronephrosis (GH) is an uncommon urological disease predominantly identified and treated in infancy or childhood [1,2]. GH is defined as a kidney containing more than 1 l of urine in its collecting system or weighing more than 1.6 % of the patient's body weight. Radiologically, it may occupy a hemiabdomen, cross the midline, or measure at least five vertebral lengths in children. In some cases, especially with large masses, the affected kidney may not be visualized on CT, which can complicate the diagnosis. GH typically manifests as an obstruction of the ureteropelvic junction, although additional causes include urolithiasis, renal ectopia, ureterovesical junction obstruction, and trauma [3,4]. In advanced cases, GH may lead to symptoms such as acute abdominal pain or respiratory distress, but many cases remain asymptomatic [[5], [6], [7]]. As GH can be difficult to diagnose before surgical intervention, it may be misdiagnosed as an ovarian tumor or an intraabdominal cystic mass [8,9]. The management of large GH depends on the underlying disease and the renal function of the affected kidney [9,10]. In this study, we present a case of a striking GH that resembled an abdominal lymphatic malformation or an omental cyst in a 16-year-old girl, successfully managed with surgical intervention. This work has been reported in line with the SCARE criteria [11].

## 2 Case report

**Fig. 1:**
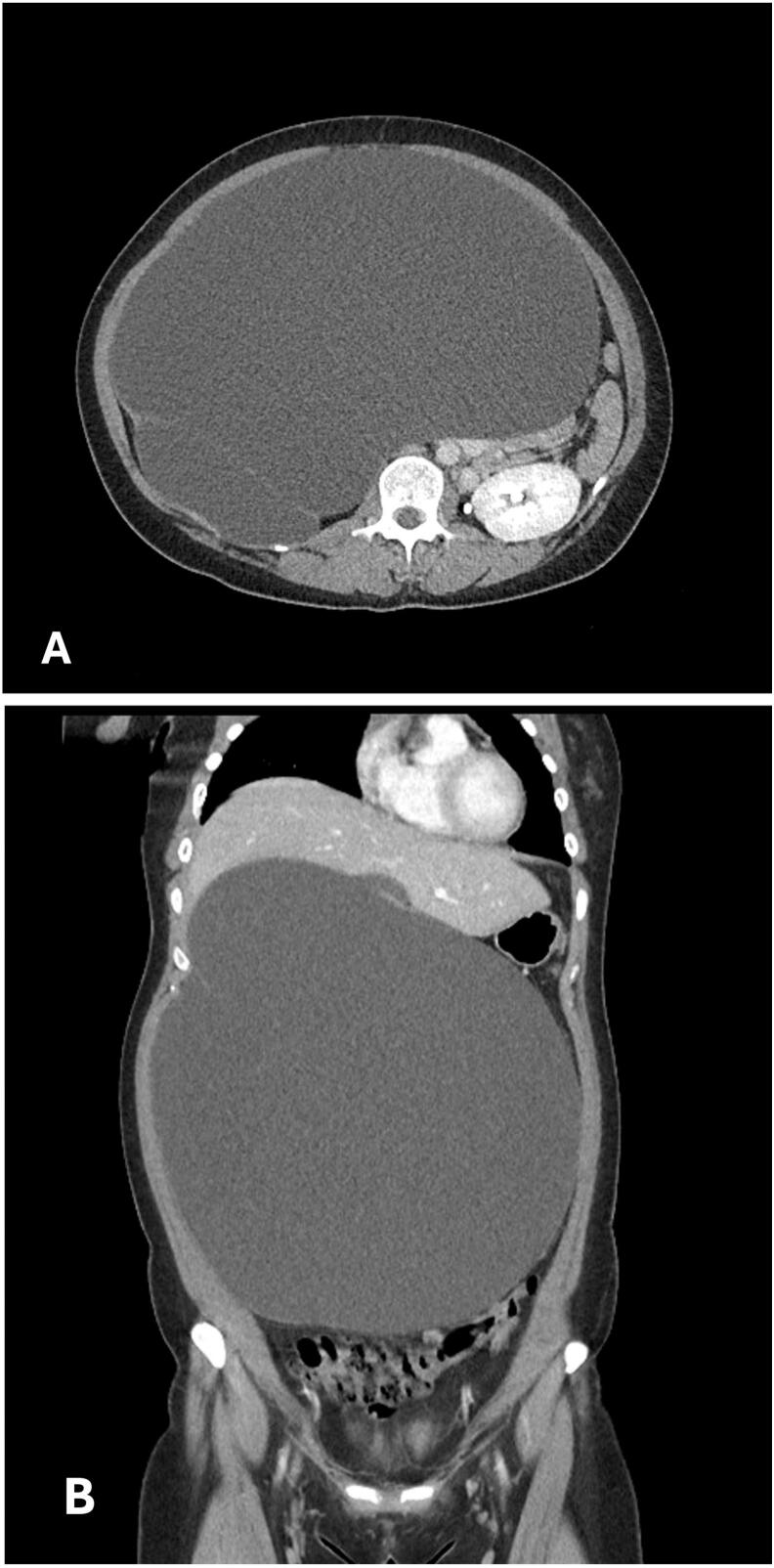
The axial (A) and coronal (B) views of an abdominal computed tomography scan reveal a large cystic mass occupying the entire abdominal cavity and displacing the bowel posteriorly, suggesting the possibility of an abdominal lymphatic malformation or an omental cyst.

**Fig. 2:**
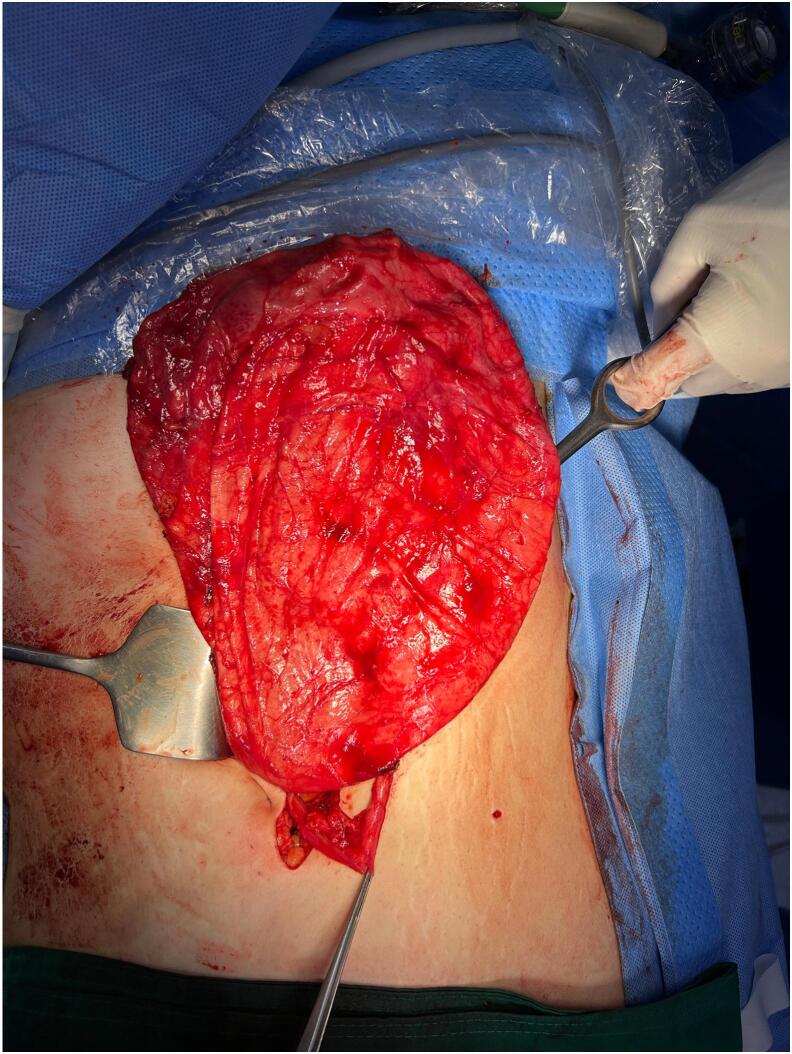
Intraoperative image of the 33-cm cystic mass arising from the right retroperitoneum.

A 16-year-old Korean girl (BMI: 25.4 kg/m^2^) presented to our hospital with a 1-month history of abdominal distention and irregular menstruation. She had no relevant medical or psychosocial history and denied symptoms related to gastrointestinal, hepatobiliary, or reproductive organs. On admission, her vital signs were as follows: blood pressure, 127/102 mmHg; heart rate, 98 beats per minute; respiratory rate, 18 breaths per minute; and body temperature, 37.0 °C. Her abdomen was markedly distended and mildly tender throughout. Initial laboratory findings were within normal limits, with a blood urea nitrogen concentration of 11.4 mg/dL (normal range, 6.0–20.0 mg/dL) and a creatinine level of 0.78 mg/dL (normal range, 0.5–0.9 mg/dL). Serum α-fetoprotein, β-human chorionic gonadotropin, and CA-125 (cancer antigen–125) levels were all normal. An abdominal computed tomography (CT) scan demonstrated a 33-cm cystic mass occupying the entire abdominal cavity and displacing the bowel posteriorly, suggesting an abdominal lymphatic malformation or an omental cyst. Both ovaries and the uterus appeared normal (Fig. 1A, B). A definitive diagnosis was not reached preoperatively. She was taken to the operating room, where an initial laparoscopic exploration was attempted under general anesthesia. The surgical team consisted of one pediatric surgeon and an experienced anesthesiologist. A 33-cm cystic mass arising from the right retroperitoneum was identified. However, technical difficulties and limited working space necessitated conversion to open surgery. A midline incision, including the umbilical trocar site, provided access to the abdominal cavity. The mass originating from the retroperitoneum was too large to be resected and decompression via needle aspiration yield approximately 8000 cc of fluid. Intraoperative fluid analysis showed a creatinine level of 33.7 mg/dL, confirming urinary origin. This result was available intraoperatively and supported the diagnosis of a urinary structure. The mass, identified as the right kidney, was resected with the right ureter without complications (Fig. 2). Histopathological examination revealed atrophied renal parenchyma and a dilated renal pelvis, consistent with GH. The patient's postoperative course was uneventful, and she was discharged on the seventh postoperative day in good condition. At the 1-year follow-up, she remained asymptomatic. Abdominal CT scan showed no abnormalities, and her serum creatinine level remained normal.

## 3 Discussion

When urinary outflow is obstructed, or urine flows back into the kidney, the collecting system may become distended, causing hydronephrosis. GH is defined as a kidney containing more than 1 l of urine in its collecting system or weighing more than 1.6 % of the patient's body weight [5]. Crooks et al. described GH as a kidney that occupies a hemiabdomen, crosses the midline, or measures at least five vertebral lengths [12]. In children, GH can account for 4 % of total body weight at birth, decreasing to about 2 % by puberty [4]. Although GH can occur at any age, the use of prenatal ultrasonography has led to most cases being identified in children. The most common cause of GH is congenital obstruction of the ureteropelvic junction due to anomalies in the pyeloureteral smooth muscle [4]. Other possible causes include vesicoureteral reflux, posterior urethral valves, urolithiasis, pyelic or ureteral tumors, trauma, retroperitoneal fibrosis, ischemia, polar or aberrant vessels, and renal or ureteral malformations (e.g., ureteral ectopia, congenital ureteral stricture, obstructive megaureter, ureteral atresia, duplicative collecting system). Given that our patient did not experience abdominal pain despite a large mass for a prolonged period, an acquired chronic obstruction of the ureteropelvic junction rather than a congenital defect is suspected.

GH often presents as an asymptomatic abdominal distention or mass that can progress to a late-stage condition [5,13]. Consequently, GH may easily be mistaken for other pathologies, such as ovarian tumors or intraabdominal cystic masses. In our case, the size and characteristics of the mass on CT scan raised suspicion for an omental or mesenteric cyst or an abdominal lymphatic malformation. Notably, the absence of the right kidney was initially overlooked, which contributed to the initial misdiagnosis, although GH was ultimately confirmed intraoperatively. Early symptoms can be nonspecific and may include nausea, constipation, dyspepsia, fever, asthenia, persistent low back pain, hematuria, recurrent urinary tract infections, and mild abdominal or flank pain [6]. Long-standing GH can lead to complications such as mechanical intestinal obstruction, hypertension, renal failure, respiratory distress, lower limb edema, obstructive jaundice, rupture, contralateral ureteropelvic junction obstruction, and malignant transformation [14].

Common imaging modalities for diagnosing GH include ultrasonography and excretory urography (either antegrade or retrograde) [9]. To distinguish GH from other potential diagnoses, abdominal CT and magnetic resonance imaging are recommended [15]. Magnetic resonance imaging is considered the gold standard for definitive diagnosis.

The differential diagnosis for GH includes massive ascites, echinococcal liver cysts, hepatobiliary cysts, mesenteric cysts, cystic renal tumors, ovarian cysts or tumors, splenomegaly, retroperitoneal tumors or hematomas, and pseudomyxoma peritonei [10].

Management of GH depends on the renal function and underlying cause [9]. Nephrectomy is the preferred approach for nonfunctioning kidneys, particularly those with more than 1 l of fluid and irreversible cortical damage. Had GH been correctly diagnosed preoperatively in our case, it is likely that laparoscopic decompression and resection could have been completed without the need for conversion to open surgery. When the renal function is uncertain, it can be assessed by 99 m‑technetium mercaptoacetyltriglycine renal scintigraphy. In functioning kidneys, treatment options include percutaneous nephrostomy, reduction pyeloplasty with nephropexy, ureterocalycostomy, calycocystostomy, or Boari flap calycovesicostomy.

## 4 Conclusion

Although GH is a rare condition in pediatric patients, we recommend including it in the differential diagnosis of large abdominal cystic masses in this population.

## Author contribution

Hyung Jun Kwon: Writing – review & editing, Writing – original draft, Methodology, Conceptualization.

Jinyoung Park: Writing – review & editing, Writing – original draft, Methodology, Conceptualization, Investigation

## Consent

Informed consent was obtained from the patient's parents for the publication of this case report. Patient confidentiality was strictly maintained throughout the study.

## Ethical approval

Ethical approval was waived by the institution's Research Ethics Committee. Case reports involving a single patient encountered during routine clinical care are exempt from ethics approval at Institutional Review Board of Kyungpook National University Hospital.

## Guarantor

Jinyoung Park

## Patient perspective

The patient's family expressed relief and satisfaction with the prompt surgical intervention and subsequent recovery. They acknowledged the critical role of timely medical care in preventing severe complications.

## Research registration number

Not applicable.

## Funding

This case report received no specific grant from any funding agency in the public, commercial, or not for profit sectors.

## Declaration of competing interest

The authors declare that there is no conflict of interest.
